# Multiscale Imaging Reveals Aberrant Functional Connectome Organization and Elevated Dorsal Striatal *Arc* Expression in Advanced Age

**DOI:** 10.1523/ENEURO.0047-19.2019

**Published:** 2019-12-18

**Authors:** Luis M. Colon-Perez, Sean M. Turner, Katelyn N. Lubke, Marjory Pompilus, Marcelo Febo, Sara N. Burke

**Affiliations:** 1 Department of Neurobiology and Behavior, University of California, Irvine, Irvine, California 92697; 2 Department of Psychiatry, University of Florida, Gainesville, Florida 32610; 3 Department of Neuroscience, University of Florida, Gainesville, Florida 32610; 4 Department of McKnight Brain Institute and College of Medicine, University of Florida, Gainesville, Florida 32610

**Keywords:** anterior cingulate cortex, functional connectivity, graph theory, prefrontal cortex, working memory

## Abstract

The functional connectome reflects a network architecture enabling adaptive behavior that becomes vulnerable in advanced age. The cellular mechanisms that contribute to altered functional connectivity in old age, however, are not known. Here we used a multiscale imaging approach to link age-related changes in the functional connectome to altered expression of the activity-dependent immediate-early gene *Arc* as a function of training to multitask on a working memory (WM)/biconditional association task (BAT). Resting-state fMRI data were collected from young and aged rats longitudinally at three different timepoints during cognitive training. After imaging, rats performed the WM/BAT and were immediately sacrificed to examine expression levels of *Arc* during task performance. Aged behaviorally impaired, but not young, rats had a subnetwork of increased connectivity between the anterior cingulate cortex (ACC) and dorsal striatum (DS) that was correlated with the use of a suboptimal response-based strategy during cognitive testing. Moreover, while young rats had stable rich-club organization across three scanning sessions, the rich-club organization of old rats increased with cognitive training. In a control group of young and aged rats that were longitudinally scanned at similar time intervals, but without cognitive training, ACC–DS connectivity and rich-club organization did not change between scans in either age group. These findings suggest that aberrant large-scale functional connectivity in aged animals is associated with altered cellular activity patterns within individual brain regions.

## Abstract (precis)

The functional connectome reflects a network architecture enabling adaptive behavior that becomes vulnerable in advanced age. The cellular mechanisms that contribute to altered functional connectivity in old age, however, are not known.

## Significance Statement

Cognitive decline is frequently observed in advanced age. Although impairments in older adults have been linked to alterations in resting-state brain connectivity, how these changes relate to the neurobiology of individual neurons is unknown. The current study reports longitudinal changes in the functional connectome of aged rats with cognitive training that were not observed in young animals with better task performance. These network alterations in old age were associated with poorer task performance and increased *Arc* expression in the dorsal striatum. This work is significant because it links functional organization of brain networks to behavioral impairments and changes within individual neurons, providing a potential bridge between invasive cell-based analyses in animal models to imaging data from human study participants.

## Introduction

Advancing age is associated with cognitive impairments that can erode one’s quality of life (Samson and Barnes, 2013; Lockhart and DeCarli, 2014). Behaviors that rely on interactions across brain networks, such as episodic memory and cognitive multitasking, appear to be particularly vulnerable to decline in older adults (Chadick et al., 2014; Fandakova et al., 2014) and animal models of aging (Hernandez et al., 2015; Gray et al., 2017). A possible reason for these cognitive impairments could be aberrant organization of the functional connectome with advancing age (Ash and Rapp, 2014; Sala-Llonch et al., 2014; Nyberg, 2017), which include decreased connectivity within the default mode network (Sala-Llonch et al., 2015; Grady et al., 2016), increased functional connectivity within the hippocampal network (Salami et al., 2014), decreased segregation between different functional networks (Chan et al., 2014; Geerligs et al., 2015), as well as increased connectivity between the anterior cingulate cortex (ACC) and other cortical structures (Cao et al., 2014b). Although it is unclear how different functional networks observed in humans map onto rodents, altered resting-state functional connectivity has also been reported for old rats (Ash et al., 2016) and middle-aged mice (Egimendia et al., 2019), indicating that there is a cross-species consensus regarding the vulnerability of brain-wide networks to advancing age.

While altered network connectivity in older adults is thought to reflect neural inefficiency or dedifferentiation (Salami et al., 2014; Sala-Llonch et al., 2015; Grady et al., 2016; Nyberg, 2017), it remains unclear how network parameters used to quantify large-scale functional connectome organization relate to age-associated neurobiological changes at the cellular level. Recent behavioral models for probing the integrity of inter-regional communication (Hernandez et al., 2015, 2017), along with advances in small animal functional MRI (fMRI; Ash et al., 2016; Colon-Perez et al., 2016) offer a unique opportunity to interrogate both large-scale functional connectome organization and cellular mechanisms of cognitive aging within the same animals, providing a critical translational link to noninvasive imaging in human study participants.

An additional advantage to working with animal models is the ability to longitudinally measure resting-state metrics of network architecture as a function of cognitive training in populations with highly controlled dietary and behavioral experiences across age groups. While working memory (WM) training in young adults (Takeuchi et al., 2017) and water maze training in young rats (Nasrallah et al., 2016) have been shown to alter functional connectivity, it is unknown whether cognitive training similarly impacts functional network architecture in aged populations.

The current study aimed to examine how cognitive training on a cognitive dual task, which required animals to perform a spatial WM and a biconditional association task (BAT) simultaneously, altered resting-state functional connectivity in young and aged rats. This behavioral paradigm is known to require interactions among prefrontal, medial temporal, and subcortical structures (Jo and Lee, 2010; Hernandez et al., 2017), and is vulnerable to decline in old age before the emergence of deficits on the hippocampus-dependent Morris water maze (Hernandez et al., 2015). Rats were scanned at three timepoints to measure longitudinal changes in brain connectivity during learning using graph theoretical analysis. The rich-club coefficient was included as an unbiased metric of network organization. The rich club refers to a set of densely and highly interconnected nodes known as hub regions in the brain. Rich-club organization is an expensive network structure (i.e., extensive connectivity and metabolic cost) that allows complex network dynamics to increase efficiency (Kaiser and Hilgetag, 2006; van den Heuvel et al., 2012). In the context of functional networks, the rich club describes an increase in participation and activation of certain active nodes into members of a functional rich club (Liang et al., 2018). Importantly, a rich club of connector hubs is believed to be more vulnerable to damage in aging due to metabolic demands (Bullmore and Sporns, 2012; Hernandez et al., 2018b).

Following the last resting-state scan, rats were assessed for the expression of the activity-dependent immediate-early gene *Arc* (Cole et al., 1989; Guzowski et al., 1999) during WM/BAT behavior to directly relate neuronal activity during the task to network connectivity patterns obtained from resting-state fMRI. Thus, the current experiments used a multiscale imaging approach that spanned from single cells to global networks and behavior to explore neural network dynamics in young and old rats in relation to cognitive training.

## Materials and Methods

### Subjects and behavioral testing

A total of 18 young (4 months old) and 22 aged (24 months old) male Fischer 344 × Brown Norway F1 (FBN) hybrid rats from the National Institute on Aging colony at Taconic Farms were used in this study. Rats were used across different cognitive training and imaging procedures, and the sample sizes for each are summarized in Table 1. Notably, the life span of FBN rats is greater than that of inbred Fisher 344 rats (Turturro et al., 1999), and many of the physical issues experienced by Fischer 344 rats are not evident in the FBN rats until they are older than 28 months (McQuail and Nicolle, 2015). Therefore, changes in performance are likely due to cognitive decline and not age-related physical impairment. A subset of five rats in each age group were shaped to run on the continuous T-maze for reward and then scanned for the network analysis at three different timepoints in relation to cognitive training on the WM/BAT. After scanning, these same rats were trained on a new WM/BAT problem set and then killed to analyze *Arc* expression. For the *Arc* experiment, a new set of objects was used for the WM/BAT because we wanted a similar time interval to pass between the final resting-state scan and the *Arc* experiment as that for separating the three resting-state scans. Given that aged rats will eventually learn the WM/BAT problem (Hernandez et al., 2015, 2018b), we selected new objects to ensure that there would still be an age-related difference in task performance after an additional 2 weeks of testing.

**Table 1:** Animal number across different experimental groups and procedures

Rat number	Experimental procedures
n = 5 young; n = 5 aged^a^	Resting-state functional MRI with cognitive training on WM/BAT and *Arc* expression analysis
*n* = 7 young; *n* = 8 aged	WM/BAT training and *Arc* expression analysis only
*n* = 5 young; *n* = 8 aged	Control rats with no cognitive training, continuous T-maze training and longitudinal resting-state scans only

^a^One aged rat was not included in the *Arc* analysis.

One aged rat reached a humane endpoint before the *Arc* compartment analysis of temporal activity with fluorescence *in situ* hybridization (catFISH) experiment and was therefore not included in the *Arc* expression analysis, but the data for this animal were included in the resting-state analysis. An additional young (*n* = 1) and aged (*n* = 1) rat were killed directly from the home cages as a negative control to ensure that nothing unexpected occurred in the colony room on the day of the experiment to increase *Arc* expression. Expression levels in these rats were low [<5% for anterior cingulate cortex and <2% for dorsal striatum (DS); data not shown]. To replicate the observation of elevated *Arc* expression in the dorsal striatum, a group of *n* = 7 young and *n* = 8 aged rats were trained on a WM/BAT for 2 weeks and then were directly killed following behavior to label *Arc* mRNA in the striatum. A final group of rats (*n* = 5 young and *n* = 8 aged) were trained to traverse a track for food reward without cognitive training and were scanned longitudinally at time intervals similar to those for the rats that were cognitively trained on the WM/BAT (baseline, at 11 d, and at 24 d). These animals served as controls to quantify the longitudinal stability of the functional connectome in the absence of cognitive training. Each rat was housed individually in a temperature- and humidity-controlled vivarium and maintained on a reverse 12 h light/dark cycle. All behavioral testing and scanning were performed in the dark phase.

All rats were allowed 1 week to acclimate to the housing facility before food restriction and initial behavioral shaping. One week after arrival, all rats were placed on restricted feeding in which 20.5 g of moist chow (1.9 kcal/g) was provided daily, and drinking water was provided *ad libitum*. Shaping began once rats reached ∼85% of their baseline weights. Baseline weight was considered to be the weight at which an animal had an optimal body condition score of 3 (with 5 being obese). Throughout the period of restricted feeding, rats were weighed daily, and body condition was assessed and recorded weekly to ensure a range of 2.5–3. The body condition score was assigned based on the presence of palpable fat deposits over the lumbar vertebrae and pelvic bones. Rats with a score <2.5 were given additional food to promote weight gain. All procedures were performed in accordance with the National Institutes of Health *Guide for the Care and Use of Laboratory Animals* and were approved by the Institutional Animal Care and Use Committee.

**Figure 1.**
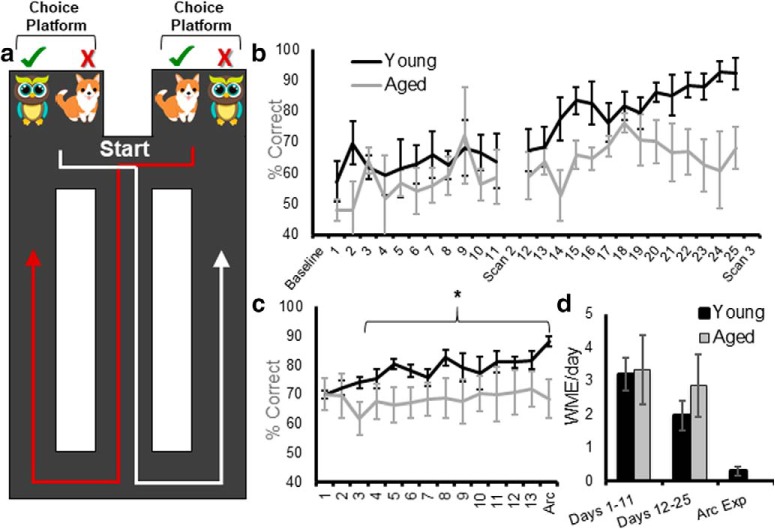
WM/BAT performance. ***a***, Schematic of the WM/BAT. Rats began testing by being placed at the location labeled start, facing away from the choice platforms and began traversing the continuous T-maze, alternating between left and right turns. After turning, before returning to the central stem, rats solved an object discrimination problem in which the correct choice (green check) was contingent on the side of the maze. On the left, the owl was rewarded, and on the right the dog was rewarded. The position of the objects over the well in a choice platform varied across trials. ***b***, Performance on WM/BAT over days of testing in young (black) and aged (gray) rats between resting-state scans. ***c***, After the third scan, rats were retrained on WM/BAT with different objects for 13 d before performing the task a final time, followed by immediate killing to label the mRNA products of the activity-dependent immediate-early gene *Arc*. ***d***, The average number of working memory errors (making two turns in the same direction rather than alternating) per day of testing in young and aged rats. The incidence of working memory errors decreased over the testing period, but did not differ by age. Error bars are ± 1 SEM, *p < 0.05.

Once rats reached 85% of their baseline weight, after ∼1 week of restriction, they began shaping on the WM/BAT track. Rats were first habituated to the testing apparatus for 10 min/d for 2 consecutive days, with Froot Loop pieces (Kellogg’s) scattered throughout the maze to encourage exploration. Following habituation, once rats were comfortable on the testing apparatus, they were trained to alternate between the left and right turns for 32 trials/d or 30 min. Every day a rat began testing by being placed on the continuous T-maze at the location labeled “start” in Figure 1*a*. This proceeded for 1 week, then rats underwent the first baseline fMRI scan. For all behavioral testing sessions before the *Arc* experiment, rats completed 32 trials/d. Thus, performance and functional connectome differences could not be accounted for by a disparity in the number of trials completed between age groups.

After the baseline scan, rats began testing on the WM/BAT of cognitive multitasking (Fig. 1*a*). Each day, testing began by placing rats at the start location facing away from the choice platforms. After traversing the central arm, rats were initially allowed to run in whichever direction they chose, but on subsequent trials were only able to perform an object discrimination task on a choice platform for reward if they alternated between trials. After making a correct turn, before returning to the center section of the maze, rats were “interrupted” with an object discrimination problem on the choice platform in which a target object could be displaced to receive a Froot Loop piece. While the same object pair was presented in both the left and right choice platforms, different objects were rewarded on the left versus right platform (owl was correct on the left platform and dog was correct on the right platform). Thus, animals had to integrate information about where in the maze they are with the object information to learn the correct biconditional association between an object and a place. Within a choice platform, the location of the target object over the left or right food well varied pseudorandomly across trials. A single trial was considered leaving the start location, making a turn, then selecting an object, and returning to the start to begin the next trial. If a rat failed to alternate correctly, objects were not placed on the choice platform and no reward was available, and this was recorded as a WM error (WME) and was not logged as a trial. The number of WMEs per day was recorded, but the percentage correct was determined from the choice of the object. During WM/BAT training, rats performed 32 trials of object discrimination testing in each training session. On the first day of testing, objects only partially covered the food reward for the first four trials per object (eight trials total) to encourage learning. Rats could begin with a trial turning in either the left or right direction, but on all subsequent trials rats had to alternate turning directions. In addition to percentage correct and WMEs, a response bias was calculated for each day of testing. This metric captures the tendency of an animal to select an object over a food well on a particular side, regardless of object identity. The response bias was calculated for each testing session as the absolute value of the difference between choosing the object over the left well versus the right well divided by the total number of trials. Thus, 1 indicates a maximal bias in which only one side was selected, and 0 is representative of no bias. Because the target object was presented over the left versus right food wells for an equal number of trials, object placement did not influence the response bias, and if a rat performed perfectly, the response bias would be 0. Rats were tested on the WM/BAT for 11 consecutive days and then given 2 d off, during which the second scanning session occurred. After the scans were completed, rats were tested for another 14 d and were scanned for a third and final time. After the last scan, rats were retrained on the WM/BAT with a new set of objects for the *Arc* catFISH experiment.

In the second group of rats, animals were food restricted and shaped to get familiar with the maze using the same procedures described above, and then were given a baseline resting-state fMRI scan. After the baseline scan, these rats then traversed the maze for a Froot Loop reward for 32 traversals/d and were scanned after 11 and 24 d of maze running. The rats in this experiment served as a control group that did not undergo cognitive training.

### Functional magnetic resonance imaging

Rats were imaged under isoflurane (1.5%) sedation (delivered in 70% N_2_/30% O_2_ at 0.1 L/min). Inhalation anesthetic agents (e.g., isoflurane) are preferred for longitudinal fMRI experiments over intravenous injectable anesthetic agents because of a better control over blood levels of the sedative, fast recovery, and lower mortality rates in rats. Important to the present study, several studies have confirmed BOLD activation patterns at low levels of anesthesia in rats (Masamoto et al., 2007; Kannurpatti et al., 2008; Kim et al., 2010; Williams et al., 2010). Isoflurane induces dose-dependent vasodilation; thus, functional experiments must be ideally performed under doses of <2% (i.e., a fixed concentration between 1 and 1.5%) as was done in the current study (fixed at 1.5%). Even in human neuroimaging studies, general anesthesia (sevoflurane) has been shown to not prevent the measurement of BOLD activity and general connectivity (Riehl et al., 2018). Spontaneous breathing was monitored during MRI acquisition (SA Instruments & Systems). Body temperature was maintained at 37–38°C using a warm water recirculation system. Heads were stabilized using a bite bar and foam side padding, and the surface coil was placed over the heads of the rats (providing further restrictions to movement). The motion correction is implemented as a default step since minor head movement is always possible, although our prior work and the work in this study show no evidence of any gross movement (Febo and Ferris, 2014; Colon-Perez et al., 2016; Orsini et al., 2018).

In the group of rats that received cognitive training, a resting-state fMRI dataset was collected with an 11.1 T Bruker MRI system (Magnex Scientific). The system is a Bruker AV3 HD console/Paravision 6.01 with a volume transmit (85-mm-inner diameter quadrature coil) and a four-channel phase-array receive coil (Rapid Rat Phase Array). All 10 rats were scanned over the span of 2 months in three scanning sessions: before cognitive training (baseline scan; *n*_young_ = 5, *n*_old_ = 5), following 11 d of training (scan 2), and after an additional 2 weeks of training (scan 3). A one-shot spin-echo echoplanar imaging (EPI) sequence was acquired with the following acquisition parameters: repetition time (TR) = 2000 ms; echo time (TE) = 15 ms; 300 repetitions for a total acquisition time of 10 min (an image was acquired every 2 s); field of view (FOV) = 25.6 × 25.6 mm^2^; 20 slices 1 mm thick; and data matrix = 64 × 64. The voxel size was 0.4 mm in place, which is within the range of geometric parameters of most rat fMRI studies. Anatomic scans for image overlay and reference-to-atlas registration were collected using a fast spin-echo sequence with the following parameters: TR/TE_eff_ = 4500/48 ms; RARE (rapid acquisition with relaxation enhancement) factor = 16; number of averages = 6; FOV = 25.6 × 25.6 mm^2^; 20 slices 1.0 mm thick; and data matrix = 256 × 256.

Because of technical issues with the four-channel phase-array receive coil that emerged after the completion of the cognitive training experiment, resting-state fMRI scans for the rats that did not receive cognitive training were collected on a 4.7 T/33 cm horizontal magnet (Magnex Scientific) with an 11.5-cm-diameter gradient insert (670 mT/m maximum gradient strength at 300 A and a 120 μs rise time; Resonance Research) and controlled by VnmrJ 3.1 software (Agilent). A quadrature transmit/receive radio frequency (RF) coil tuned to 200.6 MHz 1H resonance was used for B1 field excitation and RF signal detection (Air MRI). Functional images were collected using a two-shot spin-echo EPI sequence with the following parameters: TE = 50 ms; TR = 1 s; 32.5 × 32.5 mm in plane (voxel size = 0.5 mm); 12 slices with 1.5 mm thickness per slice; data matrix = 64 × 64. A total of 300 repetitions was collected per EPI scan (10 min), with two scans per rat. Anatomic scans for image overlay and reference-to-atlas registration were collected using a fast spin-echo sequence (TE = 45 ms; TR = 2 s; echo train length = 8; number of averages = 10; data matrix = 256 × 256) in the same space as the EPI scan.

### Image processing

Image processing and network calculations from functional connectivity-based graphs were as previously implemented and reported for mouse brain (Colon-Perez et al., 2019), rat brains (Orsini et al., 2018), and Marmoset monkey brains (LaClair et al., 2019). Brain masks were drawn manually over high-resolution anatomic scans using segmentation tools in itk-SNAP (www.itksnap.org). The masks were used to crop images and remove nonbrain voxels. The cropped brain images were aligned with a rat brain template using the FMRIB Software Library linear registration program *flirt* (Jenkinson et al., 2002), using previously published parameters (Colon-Perez et al., 2016). Registration matrices were saved and used to subsequently transform functional datasets into atlas space for preprocessing and analysis. Slight displacements in individual images over the series of 300 images and slice-timing delays were corrected, and time series spikes were removed using Analysis of Functional NeuroImages (AFNI; Cox, 1996). Linear and quadratic detrending, spatial blurring, and intensity normalization were also performed. Six head motion parameters and cerebroventricular and white matter signals were removed from all datasets. A voxelwise temporal bandpass filter (between 0.01 and 0.1 Hz) was applied to remove brain signals that contain cardiac and respiratory frequencies.

Time series fMRI signals were extracted from each region of interest (ROI) based on the atlas-guided seed location (150 total areas, divided equally in left and right representations of each region). This is a segmentation atlas based on the Paxinos and Watson (1998) atlas of the rat brain. It was developed by Ekam imaging (Northeastern University, Boston, MA; Yee et al., 2015) and has been used in a number of previous publications (Febo et al., 2009; Nephew et al., 2009, 2018; Colon-Perez et al., 2018). Signals were averaged from voxels in each ROI (Colon-Perez et al., 2016). Voxelwise cross-correlations were conducted to create correlation coefficient (Pearson *r*) maps. The first nine images in each functional time series were not used in the cross-correlation step. Pearson *r* maps were subjected to a voxelwise *z*-transformation. The AFNI *3dClustSim* program was used to determine the adequate cluster size for a given uncorrected *p* value. The resultant voxel cluster size at *p* ≤ 0.05 was used to control the level of false-positive rates in the final composite statistical maps.

### Network analysis

Brain connectivity networks were analyzed using the Brain Connectivity Toolbox for Matlab (Rubinov and Sporns, 2010), as previously reported (Colon-Perez et al., 2018; Orsini et al., 2018). Symmetrical connectivity graphs with a total 11,175 matrix entries were first organized in Matlab [graph size = *n*(*n* − 1)/2, where *n* is the number of nodes represented in the graph of 150 ROIs]. The *z*-score values of the graphs were thresholded for each subject to create matrices with equal densities (e.g., *z* values in the top 15% of all possible correlation coefficients). Matrix *z* values were normalized by the highest *z*-score, such that all matrices had edge weight values ranging from 0 to 1. Node strength (the sum of edge weights), clustering coefficient (the degree to which nodes cluster together in groups), average shortest path length (the potential for communication between pairs of structures), and small-worldness (the degree to which rat functional brain networks under study deviate from randomly connected networks) were calculated for these weighted graphs (Newman, 2003; Boccaletti et al., 2006; Saramäki et al., 2007). Brain networks were visualized using BrainNet (Xia et al., 2013). The 3D networks were generated with undirected edge weights *E*_undir_ ≥ 0.3. In these brain networks (or rat brain connectomes), the node size and color were scaled by the node strength and edges were scaled by *z*-scores. Subnetworks with high node strength (after retaining ≥15% top graph density per subject) were further analyzed to investigate the potential effects of cognitive training. This was based on the qualitative observation that in the aged rats the representation of nodes with high strength and high edge degree (*k*) changed between the baseline and subsequent scans. Critically, the experimenters who conducted the connectivity analyses were completely blind to behavioral variables. Finally, as an unbiased measure of network organization, the rich-club coefficient was calculated as the number of edges that connect nodes with degree *k* or higher. This measures the extent to which high-edge nodes are more likely to be connected to each other.

### Tissue collection and *Arc* catFISH

To investigate potential age-related differences in neuron activity during WM/BAT performance, we trained young and aged rats on a new problem set for 13 d. On the 14th day of testing, rats performed both the WM/BAT and a control spatial alternation task in which a food reward was randomly placed in a food well on the choice platform. The reward was not covered by an object, and rats did not have to perform the discrimination problem in this control task. After this first epoch of behavior, rats were placed in their home cages for a 20 min rest. Following the rest, rats performed a second epoch of behavior for 5 min. All rats performed one epoch of WM/BAT and one epoch of spatial alternation in counterbalanced order. Immediately following the second epoch of behavior, rats were placed into a bell jar containing isoflurane-saturated cotton (Abbott Laboratories), which was separated from the animal by a wire mesh shield. Animals lost righting reflex within 30 s of being placed within the jar and were immediately killed by rapid decapitation. Tissue was extracted and flash frozen in Acros Organics 2-methyl butane (Thermo Fisher Scientific) chilled in a bath of dry ice with 100% ethanol (at approximately −70°C). One additional rat in each age group was killed directly from the home cage as a negative control during the experiment to ensure that disruptions within the colony room do not lead to robust nonexperimental behaviorally induced *Arc* expression. Tissue was stored at −80°C until cryosectioning and processing for fluorescence *in situ* hybridization to label the mRNA products of the immediate-early gene *Arc* for catFISH.

Tissue was sliced at 20 μm thickness on a cryostat (model HM550, Microm) and thaw mounted on Superfrost Plus slides (Thermo Fisher Scientific). FISH for the immediate-early gene *Arc* was performed as previously described (Guzowski et al., 1999). Briefly, a commercial transcription kit and RNA labeling mix (catalog #11277073910, lot #10030660, Ambion) were used to generate a digoxigenin-labeled riboprobe using a plasmid template containing a 3.0 kb *Arc* cDNA (Steward et al., 1998). Tissue was incubated with the probe overnight, and *Arc*-positive cells were detected with antidigoxigenin–HRP conjugate (catalog #11207733910, lot #10520200, Roche Applied Science). Cy3 (Cy3 Direct FISH, PerkinElmer) was used to visualize labeled cells, and nuclei were counterstained with DAPI (Thermo Fisher Scientific). The subcellular localization of *Arc* mRNA can be used to determine which neuronal ensembles across the brain were active during two distinct episodes of behavior. *Arc* is first transcribed within the nucleus of neurons 1–2 min after cell firing. Importantly, *Arc* mRNA translocate to the cytoplasm ∼15–20 min after cell firing, which allows for cellular activity during 2 epochs of behavior, separated by a 20 min rest to be represented within a single neural population (Guzowski et al., 1999).

The *z*-stack images were collected by fluorescence microscopy (Keyence) in increments of 1 μm. Four images (two from superficial layers and two from deep layers; see Fig. 6*a*) were taken from the ACC of both the left and right hemispheres from three different tissue sections for a total of 24 images for each rat. Six images (three from medial and three from lateral) were taken from both hemispheres of the DS for a total of 36 images per rat. The percentage and subcellular location of *Arc*-positive cells were determined by experimenters blind to age and order of behavioral tasks using ImageJ software with a custom-written plugin for identifying and classifying cells. Nuclei that were not cut off by the edges of the tissue and only those cells that were visible within the median 20% of the optical planes were included for counting. All nuclei were identified with the *Arc* channel off, so as to not bias the counter. When the total number of cells in the *z* stack were identified, the *Arc* channel was turned on to classify cells as positive for nuclear *Arc*, cytoplasmic *Arc*, both nuclear and cytoplasmic *Arc*, or negative for *Arc*. A cell was counted as *Arc* nuclear positive if one or two fluorescently labeled foci could be detected above threshold anywhere within the nucleus on at least four consecutive planes. A cell was counted as *Arc* cytoplasmic positive if fluorescent labeling could be detected above background surrounding at least one-third of the nucleus on two adjacent planes. Cells meeting both of these criteria were counted as *Arc* nuclear and cytoplasmic positive.

Neural activation patterns during the WM/BAT and spatial alternation tasks were examined using the percentage of cells positive for cytoplasmic and/or nuclear *Arc* expression. A mean percentage of cells was calculated for each rat for each brain region and condition, so that all statistics were based on the number of animals for sample size, rather than images or cells. This avoids the caveat of inflating statistical power and having different dependent variables correlate with each other, which can be the case in nested experimental designs (Aarts et al., 2014). Critically, the order of behavior was counterbalanced across rats, with equal numbers in both age groups, such that cytoplasmic staining corresponded to WM/BAT task behavior and nuclear staining corresponded with alternation behavior for half of the rats and vice versa for the others. Thus, all plots showing the mean percentage of *Arc*-positive cells are in reference to the task and not to the epoch. Notably, as in other previous studies with similar behaviors (Hernandez et al., 2018b), task order did not have a significant impact on neuron activity levels.

Potential effects of age and brain region on the percentage of cells expressing *Arc* during the different behaviors (WM/BAT vs spatial alternation) were examined with factorial ANOVAs. All analyses were performed using SPSS software, version 25 or 26. Statistical significance was considered at *p* values <0.05.

## Results

### Working memory/biconditional association task performance

Rats can make two different types of errors on the WM/BAT. They can select the incorrect object, as well as take the same turn direction on adjacent trials. The latter is considered a WME. Previous studies have reported that the primary type of error made by rats is selecting the incorrect object, and that there is not a significant effect of age group on the number of WMEs (Hernandez et al., 2015, 2018b). Figure 1*b–d* summarizes the mean percentage correct for selecting the target object for young (black) and aged (gray) rats across testing days for the first (Fig. 1*b*) and the second (Fig. 1*d*) WM/BAT problem sets. Between the first and second scan, there was not a significant main effect of testing day (*F*_(10,80)_ = 0.74, *p* = 0.68), age (*F*_(1,8)_ = 1.71, *p* = 0.27), or an age by test day interaction (*F*_(10,80)_ = 0.45, *p* = 0.92). In contrast, between the second and third scans, there was a significant main effect of testing day (*F*_(13,91)_ = 3.84, *p* = 0.019). Orthogonal contrasts comparing each day of testing to performance on the day following the second scan (day 12) indicated that the percentages of correct responses were significantly greater by day 17 compared with day 12 (*p* = 0.038). There was also a trend for an age effect (*F*_(1,8)_ = 4.30, *p* = 0.07), but no significant interaction between age and test day (*F*_(13,91)_ = 1.09, *p* = 0.38). Another way to evaluate the performances of young and aged rats is to compare the total number of incorrect trials during training. Across all days of testing, the aged rats made significantly more errors than the young rats (*F*_(1,8)_ = 10.02, *p* = 0.013). Importantly, there was a significant interaction effect between age and phase of testing (days 1–11 vs days 12–25; *F*_(1,8)_ = 5.54, *p* = 0.046). *Post hoc* analysis indicated that young and aged rats made a similar number of errors before the second scan (days 1–11; *t*_(8)_ = 0.55, *p* = 0.60), but aged rats made significantly more errors before the third scan (days 12–25; *t*_(8)_ = 3.50, *p* = 0.008, corrected α = 0.025). After the third scan, rats were retrained on WM/BAT with different objects for 13 d before performing the task a final time, followed by immediate killing to label the mRNA products of the activity-dependent immediate-early gene *Arc* (Guzowski et al., 1999). Overall, rats showed significant improvements across days of testing for selecting the correct object (*F*_(13,91)_ = 2.91, *p* = 0.01). Moreover, the interaction effect between age and test day was statistically significant (*F*_(13,91)_ = 2.14, *p* = 0.02), with aged rats performing similar to young rats on the first 2 test days, but was significantly worse on the final day (*F*_(1,7)_ = 5.29, *p* = 0.05). Thus, across two different WM/BAT problem sets, aged rats performed worse than young animals.

Consistent with previous data, there was not a significant effect of age group on the number WMEs (Hernandez et al., 2015). Figure 1*d* shows the mean number of WMEs per day during the testing sessions between resting-state scans and for the *Arc* experiment. Although the rate of WMEs did not vary by age group (*F*_(1,8)_ = 0.24, *p* = 0.64), it did significantly decrease across testing (*F*_(2,16)_ = 14.31, *p* = 0.001). Importantly, the rate of WMEs across testing session did not significantly interact with age group (*F*_(1,16)_ = 0.44, *p* = 0.65), suggesting that this type of performance error could not account for the different effects of cognitive training on the functional connectome between young and old rats.

**Figure 2.**
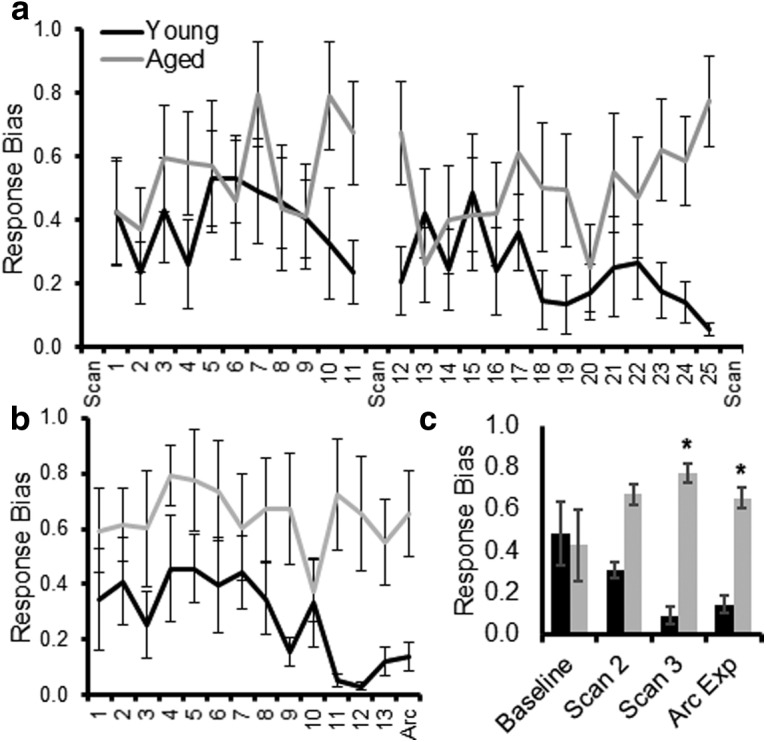
Response biases during WM/BAT training. ***a***, The response bias index quantifies the tendency of a rat to select an object on one side (left vs right) regardless of the object identity and choice platform on the maze. A value of 0 is no bias, and a value of 1 is a complete bias to one side. Most rats show a response bias that decreases as they start to perform better. Aged rats have a tendency to retain a response bias across more days of testing. The response bias is plotted across testing days between resting-state fMRI scans. ***b***, The response bias is shown across days of testing before and on the day of the *Arc* experiment. Overall, the aged rats showed a significantly greater response bias than the young rats. ***c***, The response bias of young and aged rats on the day immediately following the baseline scan, the days before scans 2 and 3, and on the day of the *Arc* experiment. Aged rats had a significantly greater response bias compared with young rats before scan. Error bars are ± 1 SEM, *p < 0.05.

Previous studies have reported that before animals learn an object discrimination problem, they show a significant response bias by selecting an object over a food well on a particular side (left vs right) regardless of object identity (Lee and Byeon, 2014; Hernandez et al., 2015; Johnson et al., 2017a). This innate response bias must be overcome before animals will learn the biconditional rule (Lee and Byeon, 2014). Figure 2*a* shows the mean response biases for young and aged rats across testing sessions between resting-state scans. The response bias did not significantly change across testing days 1–11 (*F*_(10,80)_ = 0.81, *p* = 0.62), and there was not a significant difference in the response bias between young and aged rats between scans 1 and 2 (*F*_(1,8)_ = 0.81, *p* = 0.19). Before scan 2, the interaction effect of age and testing day also did not reach statistical significance (*F*_(10,80)_ = 0.88, *p* = 0.56). Between scans 2 and 3, across testing days 12–25, the response bias also did not significantly change as a function of test day (*F*_(13,104)_ = 1.03, *p* = 0.43), and there was not a significant effect of age (*F*_(1,8)_ = 2.92, *p* = 0.13). The interaction effect between testing day and age, however, did reach statistical significance (*F*_(13,104)_ = 2.70, *p* < 0.01). This was due to the young and aged rats having a similar response bias across days 12–17, but with the young rats having a decreasing bias on the later testing days. This decreasing bias as a function of testing was not observed in aged rats. Figure 2*b* shows the mean response bias for young and aged rats while being trained on the new WM/BAT problem for the *Arc* experiment. In this phase of cognitive training, the response bias did not change as a function of testing day (*F*_(13,91)_ = 1.23, *p* = 0.27). Although the interaction effect of age and test day was also not significant (*F*_(13,91)_ = 0.99, *p* = 0.47), the aged rats overall had a significantly higher response bias relative to young animals (*F*_(1,7)_ = 6.41, *p* = 0.04). To further examine how the response bias might relate to experimental stage, the biases on days adjacent to resting-state scans and during the *Arc* experiment were also examined. Figure 2*c* shows the mean response biases on the first day of testing after the baseline scan, on the days before scans 2 and 3, and on the day of the *Arc* experiment for young and aged rats. Across these critical experimental days, there was not an overall significant effect of day on the response bias (*F*_(3,21)_ = 0.51, *p* = 0.68), but aged rats had a significantly larger response bias relative to the young animals (*F*_(1,7)_ = 49.83, *p* = 0.0001), which is consistent with previous reports (Hernandez et al., 2015; Johnson et al., 2017a). The interaction effect between age and day was also significant (*F*_(3,21)_ = 10.60, *p* = 0.014). *Post hoc* analyses indicated that young and aged rats showed comparable response biases on the first day of testing (*t*_(8)_ = 0.25, *p* = 0.81), a trend toward an age effect before scan 2 (*t*_(8)_ = 1.88, *p* = 0.09), and a significantly higher response bias in aged rats relative to young rats before scan 3 (*t*_(8)_ = 4.66, *p* = 0.002; Bonferroni corrected α = 0.05/4 = 0.0125) and during the *Arc* experiment (*t*_(7)_ = 3.35, *p* = 0.012; Bonferroni corrected α = 0.05/4 = 0.0125). These data suggest that all rats began testing with a response bias, but the young animals abandoned this suboptimal strategy early in training. In contrast, the aged rats perseverated and continued to use this strategy across testing sessions.

### The effect of cognitive training on resting-state connectivity

**Figure 3.**
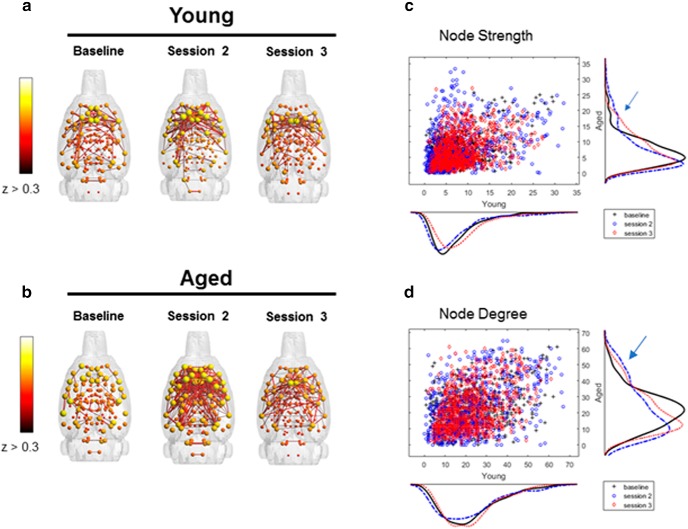
Connectivity patterns by scanning session and age group. ***a***, ***b***, Connected modules with edges *z* > 0.3 for young (***a***) and aged (***b***) rats across scan sessions. Connectivity indices indicate larger network engagement between baseline and the second and third scanning sessions in aged rats (blue arrow), but not young rats. ***c***, ***d***, This is indicated by more nodes with node strength >15 (***c***) and node degree >40 (***d***).

Over the past decade, graph theoretical approaches have been widely used to quantify functional brain networks (Bullmore and Sporns, 2009, 2012; Ash and Rapp, 2014). This analytical approach models the brain as a complex network composed of nodes (i.e., brain regions) and edges (i.e., functional correlations) connecting the nodes (Bullmore and Sporns, 2009). Figure 3, *a* and *b*, shows the 3D brain networks with functional edges with *z*-scores >0.3 in young and aged rats across scanning sessions. Quantified metrics for global network connectivity included node strength, node degree, path length, and clustering coefficient, which are defined in Table 1. None of these variables were significantly affected by scanning session or age, and the scanning session by age interaction did not reach significance (Table 2, statistical summary). These findings are consistent with a previous study reporting that resting-state networks are consistent over time (Iordan et al., 2017).

**Table 2:** Quantification of global internode connectivity patterns

Variable	Scanning session	Age	Scanning session × age interaction
Node strength	*F*_(2,29)_ = 1.06, *p* = 0.36	*F*_(1,29)_ = 0.52, *p* = 0.47	*F*_(2,29)_ = 0.21, *p* = 0.81
Node degree	*F*_(2,29)_ = 0.50, *p* = 0.61	*F*_(1,29)_ = 2.00, *p* = 0.17	*F*_(2,29)_ = 0.50, *p* = 0.61
Path length	*F*_(2,29)_ = 0.86, *p* = 0.44	*F*_(1,29)_ = 0.59, *p* = 0.45	*F*_(2,29)_ = 0.10, *p* = 0.90
Clustering coefficient	*F*_(2,29)_ = 0.54, *p* = 0.59	*F*_(1,29)_ = 0.11, *p* = 0.73	*F*_(2,29)_ = 0.41, *p* = 0.67

**Figure 4.**
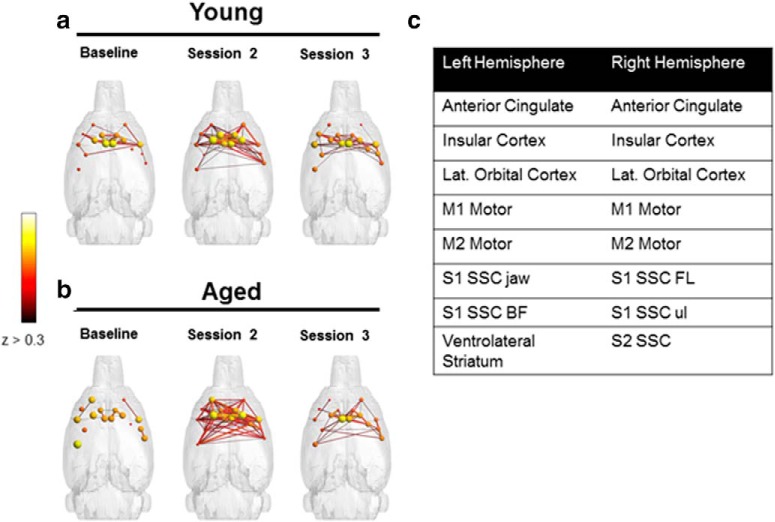
Nodes with increased strength. Sixteen nodes with strength >15 during scan 2 in aged rats were identified from the distribution shown in Figure 1. ***a***, ***b***, Connectivity patterns of these nodes for the young (***a***) and aged (***b***) groups. The table in ***c*** lists the regions by hemisphere that correspond to these nodes.

**Figure 5.**
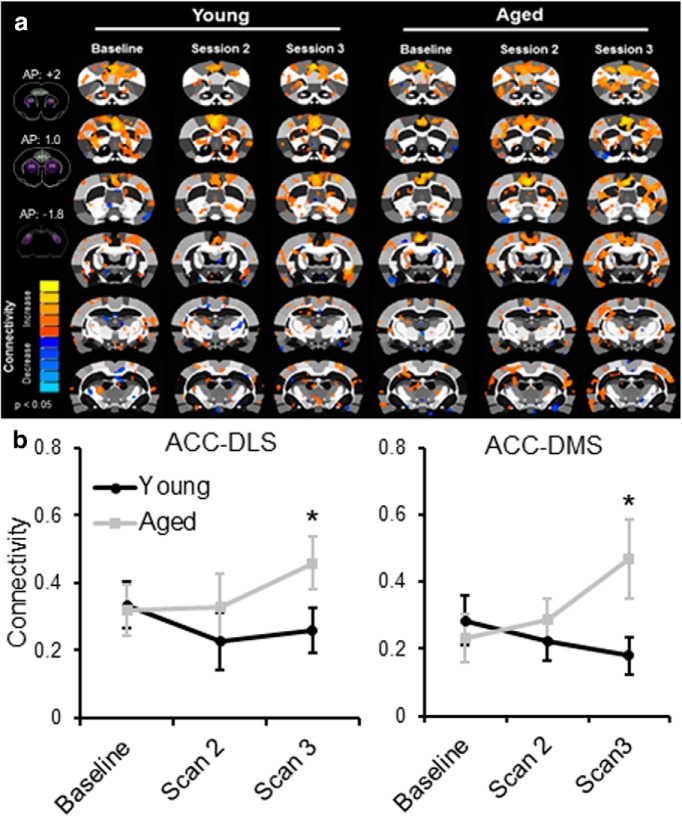
Seed analysis of ACC connectivity. ***a***, Connectivity with ACC in young (left) and aged (right) rats as a function of scan session. The insets on the left show the locations of the ACC (green shaded) and DS (purple shaded) for bregma locations anterior to posterior +2 to −1.8. ***b***, Connectivity between the ACC and DS as a function of scan session for the lateral (left) and medial (right) subregions in young (black) and aged (gray) rats. The main effects of scan session (*F*_(2,64)_ = 1.69, *p* = 0.19), age (*F*_(1,32)_ = 3.67, *p* = 0.07), and subregion of the striatum (*F*_(1,32)_ = 1.15, *p* = 0.29) did not reach statistical significance. The interaction effect between age and scan session was significant, however (*F*_(2,64)_ = 3.75, *p* = 0.03). *Post hoc* analysis indicated that there were no significant age differences between ACC–DS connectivity during the baseline scan (*p* = 0.63) and scan 2 (*p* = 0.26), but the aged rats had significantly higher connectivity relative to young rats during the third scan (*p* = 0.004). Error bars are ± 1 SEM, *p < 0.05.

Figure 3, *c* and *d*, shows the distributions of node strength and node degree, respectively, for the young and aged rats. It is qualitatively evident from these distributions that in the aged rats there was a change across scanning sessions in patterns of connectivity that reflected an increased representation of nodes with high strengths (*s* > 15; Fig. 3*c*) and high edge degrees (*k* > 40; Fig. 3*d*) between the baseline and subsequent scan sessions (Fig. 3*c*,*d*; blue arrows). From this distribution, 16 nodes with strength values >15 during the second scan session in the aged rats were identified. Figure 4 shows the connectivity between these nodes in young (Fig. 4*a*) and aged (Fig. 4*b*) rats across scanning sessions, as well as the associated brain regions for these 16 nodes (Fig. 4*c*). The ACC was identified as a node with higher strength values as a function of WM/BAT training in both hemispheres. Because there are known age-related physiologic changes within this region (Insel et al., 2012; Insel and Barnes, 2015), and morphologic differences in this structure are implicated in successful aging (Rogalski et al., 2012), we used the ACC as a seed to quantify functional connectivity between this region and other nodes in relation to scan session (Fig. 5*a*). This analysis revealed that the functional connectivity between ACC and DS in the both the lateral [dorsolateral striatum (DLS)] and medial [dorsomedial striatum (DMS)] subregions changed as a function of scan session and age (Fig. 5*b*). Although ACC–DLS and ACC–DMS connectivity was measured separately for each hemisphere, there was no significant main effect of left versus right hemisphere (*F*_(1,32)_ = 0.001, *p* = 0.97), and these were averaged together. The main effect of scan session on ACC–DS connectivity did not reach statistical significance (*F*_(2,64)_ = 1.69, *p* = 0.19). Moreover, the strength of the connectivity between the ACC and DLS versus DMS was not significantly different (*F*_(1,32)_ = 1.15, *p* = 0.29). There was, however, a trend for aged rats to have significantly higher ACC–DS connectivity compared with young animals (*F*_(1,32)_ = 3.67, *p* = 0.07). Importantly, the interaction between age and scan session was significant (*F*_(2,64)_ = 3.75, *p* = 0.03). *Post hoc* analysis indicated that there were no significant age differences between ACC–DS connectivity during the baseline scan (95% confidence interval, −0.11 to 0.17; *p* = 0.63), and scan 2 (95% confidence interval, −0.24 to 0.07; *p* = 0.26), but the aged rats had significantly higher connectivity relative to young rats during the third scan (95% confidence interval, −0.42 to −0.09; *p* = 0.004). These data are interesting in the context of the behavioral results showing that aged rats have a significantly larger response bias across training relative to the young rats (Fig. 2). It is well established that the DS is involved in response-based learning strategies (Packard and McGaugh, 1992; Gold, 2004), and aged rats may default to more response-based strategies as spatial learning becomes impaired (Barnes et al., 1980; Tomas Pereira et al., 2015). Thus, the increased ACC–DS connectivity observed here may be a network signature of the enhanced response bias seen at the behavioral level.

**Figure 6.**
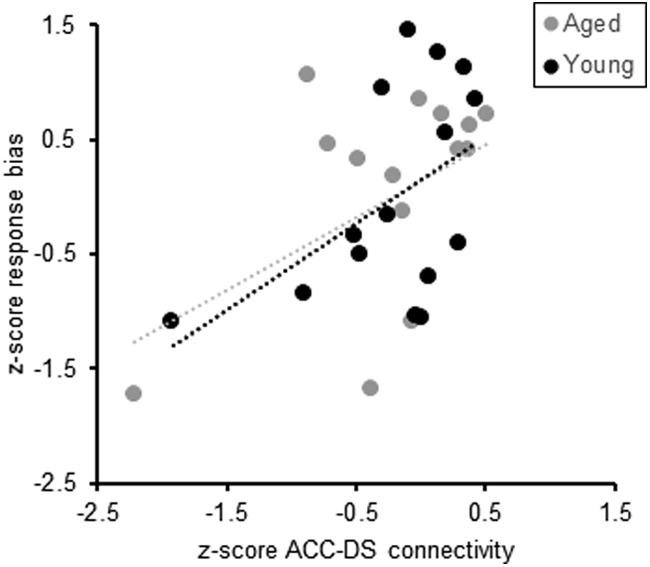
Response bias and ACC–DS connectivity. Across the three scanning sessions, the mean response bias of individual rats measured during behavior significantly correlated with ACC–DS connectivity (*R*_(29)_ = 0.48, *p* = 0.008), such that more connectivity corresponded with a higher proportion of trials in which this aberrant strategy was used. Dashed lines indicate the line of best fit for young (black) and aged (gray) rats.

To further examine the idea that enhanced ACC–DS connectivity was related to the higher response bias in aged rats with poor performance, we quantified the correlation between ACC–DS connectivity and the response bias across the three scanning sessions. To avoid detecting a spurious correlation due to age differences, all variables were transformed to z-scores calculated for the different age groups separately. This approach accounts for the tendency to detect spurious correlations due to age differences rather than an actual relationship between variables (Gerrard et al., 2008). Figure 6 shows the response bias at three different timepoints across training measured during days adjacent to the scanning session plotted as a function of ACC–DS connectivity. Across the three scanning sessions, ACC–DS connectivity accounted for a significant variance in the normalized mean response bias of individual rats (*R*_(27)_ = 0.48, *F*_(1,27)_ = 8.19, *p* = 0.008), such that more connectivity corresponded with a higher proportion of trials in which this aberrant strategy was used. Note that the correlations were similar when both aged groups were considered separately (*R* = 0.47 for young rats and *R* = 0.49 for aged rats), and when age was entered as a covariate into the model, it did not account for a significant amount of the variance in the relationship between normalized ACC–DS connectivity and normalized response bias (*R* = 0.01, *p* = 0.95). This observation suggests that in the young animals, the decreasing response bias across cognitive testing may have been facilitated by a concurrent decrease in ACC–DS connectivity. In contrast, the abilities of aged rats to inhibit a response-based strategy may have been hindered by ACC–DS connectivity that increased across testing.

**Figure 7.**
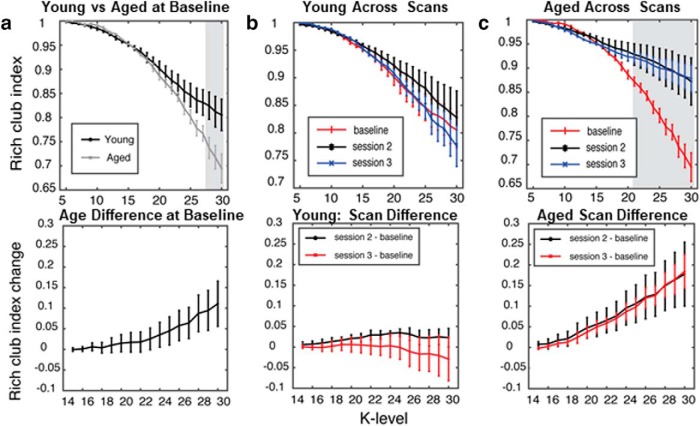
Rich-club organization increases with cognitive training in aged rats. ***a***, Top, Rich-club participation index between young and aged rats, at the baseline scan (shaded area K > 27 and *p* < 0.05). Bottom, The overall difference in increase between aged and young rats. ***b***, Top, Rich-club participation index for young rats. Bottom, The difference between scanning sessions. No changes in any of the sessions or any *k* level were observed. ***c***, Top, The rich-club participation in the aged group increased following cognitive training for large *k* levels (shaded area, *k* > 21; *p* < 0.05). Bottom, The overall difference in increase for both sessions. Error bars are ± 1 SEM, and shaded regions indicate *p* < 0.05.

Because of the potential presence of a subnetwork of “hub” nodes driving the network patterns in older rats, we next assessed the “rich-club” index. Rich-club indices were calculated for young and aged rats as a function of scanning session. Figure 7 shows the rich-club organization in young and aged rats at baseline (Fig. 7*a*) and after cognitive training (Fig. 7*b*,*c*) as a function of *k*. Similar to previous reports from human study participants (Cao et al., 2014a), the functional rich-club architecture during the baseline scan was significantly reduced in aged rats relative to young rats for rich-club indices quantified at high edge degrees (*k* level > 27, *p* < 0.05). The young rats had stable functional rich-club organization across all scans (Fig. 7*b*), which has previously been reported (Liang et al., 2018). Interestingly, in the aged rats there was a significant increase in rich-club participation after the baseline scan (Fig. 7*c*). Starting after the first training period (11 d), rich club for indices for edge values >20 displayed a significant main effect of scanning session (*F*_(2,29)_ > 3.4, *p* < 0.05, for all comparisons), but not of age (*F*_(1,29)_ < 0.55, *p* > 0.5, for all comparisons). The interaction effect of age and scanning session, however, was statistically significant for large *k* levels (*k* > 28; *F*_(2,29)_ > 3.80, *p* < 0.05, for all comparisons). As evident in Figure 7, *b* and *c*, the significant interaction between scan session and rich-club participation was due to the aged rats having greater rich-club indices after cognitive training, while the young rats did not show a change.

### Behaviorally induced expression of the immediate-early gene *Arc*

**Figure 8.**
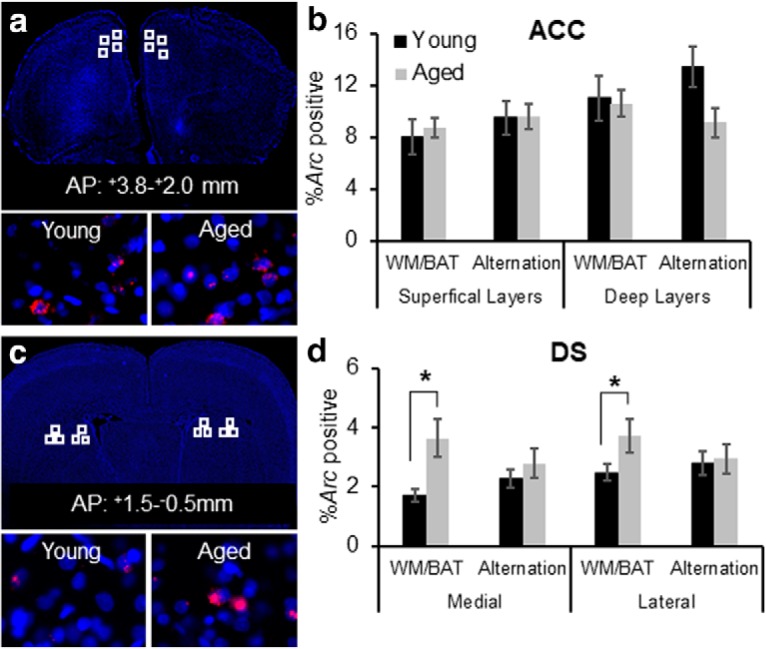
Neuronal activation during WM/BAT and spatial alternation in young and aged rats. ***a***, A DAPI-stained section showing regions that were sampled (top, white squares) within the ACC. Bottom panels show representative *Arc* labeling (red) in a young rat (left) and an aged rat (right). ***b***, Percentage of *Arc*-positive cells in the ACC corresponding to transcription during WM/BAT or the alternation task in young (black) and ages (gray) rats. There was not a significant main effect of task, age, or interaction effect on the *Arc* expression patterns. ***c***, A DAPI-stained section showing regions within the DS that were sampled for analysis of *Arc* expression (top, white squares). Bottom panels show representative images labeled for *Arc* mRNA from a young rat (left) and an aged rat (right). ***d***, Percentage of *Arc*-positive cells in the DS corresponding to transcription during WM/BAT or the alternation task. The aged rats had significantly more cells that were positive for *Arc* compared with the young animals (*F*_(1,28)_ = 5.58, *p* < 0.03). The interaction effect between age and task was also significant (*F*_(1,28)_ = 13.64, *p* < 0.001), such that aged rats had more cells than young rats that transcribed *Arc* during WM/BAT (*p* < 0.001), but this same difference was not observed during the alternation task (*p* = 0.43). Error bars are ± 1 SEM. *p < 0.05.

Because of the elevated response bias of aged rats (Fig. 2*b*,*c*), and the observation that the old animals showed an increase in ACC–DS connectivity as a function of cognitive training, we focused our *Arc* catFISH analysis on the ACC and DS. Figure 8*a* shows the region of ACC that was imaged and representative examples of *Arc* labeling in a young and an aged rat. The percentage of ACC neuron activation during the WM/BAT and spatial alternation task are shown for young and aged rats in Figure 8*b*. Repeated-measures ANOVA with the within-subject factor of task and the between-subjects factors of age group, hemisphere, and cortical layer did not detect a significant difference in the proportion of cells activated during WM/BAT versus spatial alternation (*F*_(1,28)_ = 1.53, *p* = 0.23). Additionally, none of the other between-subjects factors reached statistical significance (*F*_(2,28)_ < 3.01, *p* > 0.09, for all comparisons), nor were any of the interaction terms significant (*F*_(1,28)_ < 2.99, *p* > 0.1, for all comparisons).

Figure 8*c* shows the areas of the DS that images were collected from, as well as representative examples of *Arc* labeling in young and aged rats. To match the functional connectivity analyses, samples were taken from both the medial and lateral DS. Figure 8*d* shows the percentages of DS neurons that were positive for *Arc* during the different tasks in young and aged rats. Repeated-measures ANOVA with the within-subject factor of task and the between-subjects factors of age group, hemisphere, and subregion (medial vs lateral DS) indicated that there was not a significant main effect of task on the percentage of cells positive for *Arc* (*F*_(1,28)_ = 1.24, *p* = 0.28). The aged rats, however, had significantly more cells that were positive for *Arc* compared with the young animals (*F*_(1,28)_ = 5.58, *p* < 0.03). This age difference was observed in both the medial and lateral DS, as indicated by the lack of a main effect of subregion (*F*_(1,28)_ = 0.85, *p* = 0.37). The interaction effect between age and task was also significant (*F*_(1,28)_ = 13.64, *p* < 0.001), such that aged rats had more cells than young rats that transcribed *Arc* during WM/BAT (*p* < 0.001), but this same difference was not observed during the alternation task (*p* = 0.43). These data indicate that the enhanced DS activation in aged rats was specific to the behavioral in task in which the old animals demonstrated a deleterious response bias associated with worse performance. No other interaction effects reached statistical significance (*F*_(1,28)_ < 1.81, *p* > 0.18, for all comparisons). Due to the relatively small numbers of animals that both received longitudinal resting-state fMRI scans and were examined for *Arc* expression, we also measured WM/BAT-related *Arc* expression in the DS for a second group of rats (*n* = 7 young and *n* = 8 aged). These rats were tested over 2 weeks and then killed directly following behavior using procedures that were identical to those used in the first cohort. As with the first cohort, the aged rats performed significantly worse than the young rats (*t*_(14)_ = 3.21, *p* = 0.006) and had a significantly higher response bias (*t*_(14)_ = 2.64, *p* = 0.01). Moreover, the average percentage of *Arc*-positive cells in the DS was 3.56% for the aged rats and 2.01% for the young rats. This was comparable to the levels of *Arc* expression observed for the first cohort. Moreover, in this second cohort of animals, the aged rats had significantly more cells in the DS that were *Arc* positive compared with the young animals (*t*_(14)_ = 2.22, *p* = 0.04). Notably, the response bias index in six of the eight aged rats was >0.9 (near ceiling), which did not allow for sufficient parametric space to detect potential correlations. However, the two aged rats with lower response bias indices (0.06 and 0.25) also had levels of *Arc*-positive cells that were in the range of young animals (1.63 and 1.93%, respectively). These data confirm our initial observations and further support the idea that elevated activity in the DS is related to suboptimal response-based behavior during WM/BAT testing.

It is conceivable that behavioral differences other than the elevated response bias could account for the increased *Arc* in the DS of aged rats, such as differences in the number of trials completed or rewards received. The number of trials completed during the *Arc* experiment was compared across tasks and aged groups with repeated-measures ANOVA with the within-subject factor of task and the between-subjects factor of age group. This comparison did not detect a significant difference in the number of WM/BAT and spatial alternation trials completed (*F*_(1,7)_ = 2.31, *p* = 0.17). Moreover, the main effect of age did not reach statistical significance (*F*_(1,7)_ = 3.31, *p* = 0.11), nor was the interaction between age and task significant (*F*_(1,7)_ = 0.61, *p* = 0.46). Thus, the differences in neuron activation in DS could not be explained by animals performing a different number of trials.

**Figure 9.**
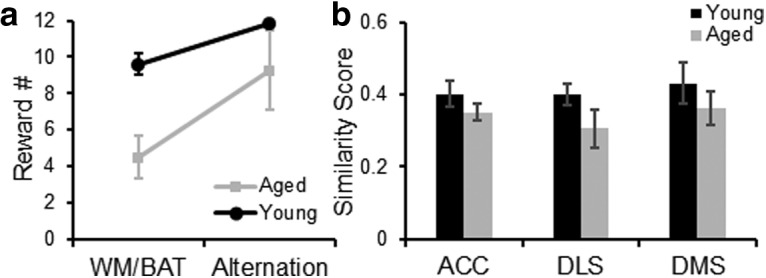
Reward expectancy and population overlap in ACC and DS. ***a***, The average number of rewards received for young (black) and aged (gray) rats during the WM/BAT and control alternation task on the day of the *Arc* experiment. Rats received more rewards during the alternation task compared with WM/BAT (*F*_(1,7)_ = 27.27, *p* = 0.001). There was also a trend for aged rats to receive fewer rewards than young rats (*F*_(1,7)_ = 5.55, *p* = 0.051) and for this to interact with task (*F*_(1,7)_ = 3.67, *p* = 0.097). ***b***, The average similarity score for young (black) and aged (gray) rats in the ACC, DLS, and DMS. The main effect of region was not significant (*F*_(2,14)_ = 0.58, *p* = 0.57). There was also not a significant main effect of age on similarity score (*F*_(1,7)_ = 2.83, *p* = 0.14), nor did the interaction effect between region and age reach statistical significance (*F*_(1,7)_ = 0.15, *p* = 0.86). Error bars are ± 1 SEM.

Another possible reason for the elevated *Arc* expression in the DS of aged compared with young rats could be a reward expectancy error from the aged rats receiving fewer rewards during WM/BAT testing compared with the young animals. Figure 9*a* shows the mean number of rewards received for the young and aged rats across the WM/BAT and control alternation task. Rats received more rewards during the alternation task compared with WM/BAT (*F*_(1,7)_ = 27.27, *p* = 0.001). There was also a trend for aged rats to receive fewer rewards than young rats (*F*_(1,7)_ = 5.55, *p* = 0.051), and for this to interact with task (*F*_(1,7)_ = 3.67, *p* = 0.097). If differences in reward experience across tasks were to account for altered expression patterns of *Arc* in the DS, then one could predict that the overlap in active ensembles across tasks would be reduced in response to differences in reward experience. To examine the extent that the active neuronal ensemble changed between the different tasks, a similarity score was calculated. As population overlap can be affected by differences in overall activity levels, similarity scores can be calculated to control for differences in activity between regions (Vazdarjanova and Guzowski, 2004). The similarity scores were compared between the ACC, DLS, and DMS (within-subjects factors of region) and the between-subjects variable of age. Because of reports that neurons in the DMS are more responsive to reward contingency and expectancy than those in the DLS (Balleine and O'doherty, 2010; Gremel and Costa, 2013; Regier et al., 2015), these regions were considered separately. Figure 9*b* shows the average similarity scores for young and aged rats for the different regions of interest. The main effect of region was not significant (*F*_(2,14)_ = 0.58, *p* = 0.57), indicating that the ACC, DLS, and DMS updated activity patterns similarly in response to performing a different task within the same environment. There was also not a significant main effect of age on similarity score (*F*_(1,7)_ = 2.83, *p* = 0.14), nor did the interaction effect between region and age reach statistical significance (*F*_(1,7)_ = 0.15, *p* = 0.86). The comparable similarity scores between age groups suggests that differences in reward experience cannot fully account for the elevated *Arc* expression in the DS in aged compared with young rats.

### Anterior cingulate cortex–dorsal striatum connectivity and rich-club organization are stable across weeks of maze traversals in the absence of cognitive training

**Figure 10.**
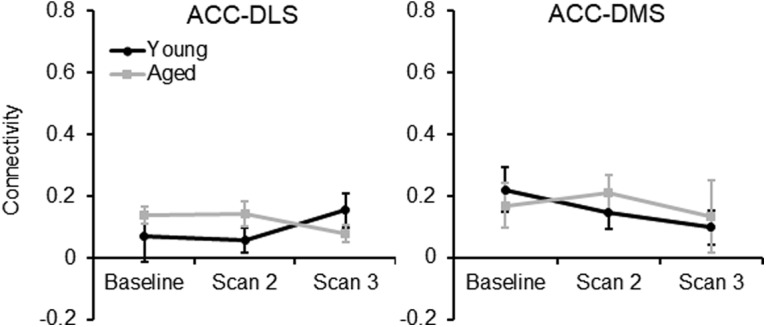
ACC–DS connectivity in untrained rats. In rats that were scanned for resting-state connectivity longitudinally after 11 and 24 d of traversing a track for reward for 32 trials, there were no significant differences between ACC–DS connectivity in the lateral (left) and medial (right) subregions across scan session (*F*_(2,88)_ = 0.39, *p* = 0.68) or age group (*F*_(1,44)_ = 0.57, *p* = 0.46). Moreover, the interaction of age and scan was not significant (*F*_(2,88)_ = 0.82, *p* = 0.44). Finally, there was no effect of DS subregion (*F*_(1,44)_ = 1.95, *p* = 0.17) and none of the other interaction terms were statistically different (*p* > 0.1 for all comparisons).

**Figure 11.**
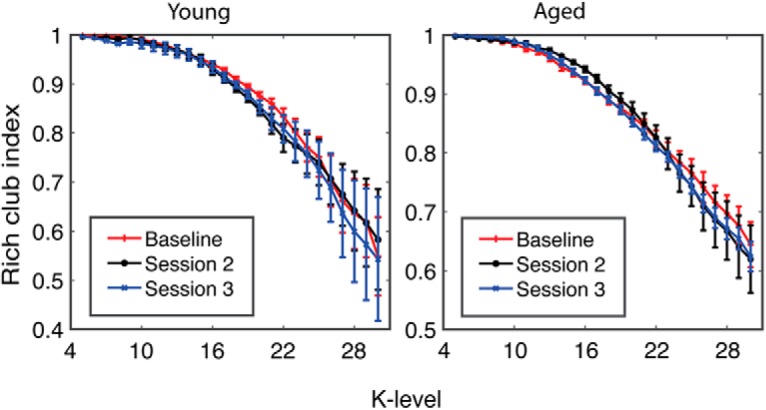
Rich-club organization does not change in untrained rats. The rich-club participation index in young (left) and aged (right) rats across the different scan sessions. Across longitudinal scans, the rich-club index did not change in either age group.

To enhance the rigor of the experiment showing that cognitive training altered the functional connectome of aged rats, but not young rats, an additional group of young and aged rats that did not perform the WM/BAT were longitudinally scanned at time intervals similar to those of the original experimental group (baseline, at 11 d, and at 24 d). These rats were trained to traverse a track for food reward for 32 trials/d to match the procedural and physical demands of the rats that received cognitive training. Figure 10 shows the connectivity between the ACC and the DSL (Fig. 10, left) and DSM (Fig. 10, right) as a function of scan session for the young and aged rats. In contrast to the rats that were cognitively trained, there was no interaction between age and scan session on the ACC–DS connectivity. Specifically, ACC–DS connectivity in the lateral (left) and medial (right) subregions did not significantly vary across scan session (*F*_(2,88)_ = 0.39, *p* = 0.68) or age group (*F*_(1,44)_ = 0.57, *p* = 0.46). Moreover, the interaction of age and scan was not significant (*F*_(2,88)_ = 0.82, *p* = 0.44). Finally, there was no effect of DS subregion (*F*_(1,44)_ = 1.95, *p* = 0.17), and none of the other interaction terms were statistically different (*p* > 0.1 for all comparisons). These data suggest that cognitive training on WM/BAT was the likely reason for enhanced ACC–DS connectivity in aged rats during scan 3. Rich-club organization was also examined in these rats. Figure 11 shows the rich-club index in young and aged rats as a function of *k* level. These values were consistent across scan session in the untrained rats in both age groups (*p* > 0.1 for all comparisons).

## Discussion

The current study used a multiscale imaging approach, integrating resting-state fMRI data with single-cell imaging of neuron activity, to determine the global network changes and cellular activity patterns in young and aged rats in relation to cognitive training. Critically, the resting-state data were acquired longitudinally as a function of training on a WM/BAT. Similar to a previous report, the aged rats were impaired on the WM/BAT relative to the young rats (Hernandez et al., 2015). Additionally, the aged rats showed an elevated response bias during testing such that they often selected an object on one side of the choice platform, regardless of their location on the maze (left vs right platform) or the object identity (Fig. 2). This suboptimal response-based strategy in aged rats has been reported in other behavioral experiments (Hernandez et al., 2015; Johnson et al., 2017b). Importantly, the ability to inhibit this response-driven strategy is dependent on the medial prefrontal cortex and is associated with performance improvements (Lee and Byeon, 2014).

Several novel findings were found in this study. First, in the aged rats there was a subnetwork that showed an increase in node strength and degree between the first scan session and the two sessions that occurred after cognitive training (Figs. 3, 4). This increase in node strength and degree occurred independent of any significant increases in cognitive performance. The increased node strength and connectivity were evident in both hemispheres of the ACC, which is a brain region vulnerable in old age (Vaidya et al., 2007; Insel et al., 2012). Moreover, anatomic variations in ACC morphology have been implicated in successful aging (Gefen et al., 2015). Interestingly, when the ACC was used as a seed for further functional connectivity analyses, both the lateral and medial subregions of the dorsal striatum were identified as regions that showed resting-state connectivity with the ACC that changed over WM/BAT testing differentially in young and old animals. In aged rats, ACC–DS functional connectivity increased as a function of cognitive training. In contrast, the young animals showed no significant change in ACC–DS functional connectivity between the baseline and subsequent scans. Despite functional distinctions between the lateral and medial subregions of the DS, with the former being more related to habits and response-driven behaviors (Balleine and O'doherty, 2010; Gremel and Costa, 2013; Burton et al., 2015; Regier et al., 2015), the enhanced ACC–DS connectivity after cognitive training in aged rats was observed in both of the subregions.

In the current study, as in other work (Hernandez et al., 2015), when rats performed poorly on the WM/BAT, they exhibited a response bias. When examined across all scanning sessions in both young and aged rats, the larger response biases were significantly correlated with enhanced ACC–DS connectivity (Fig. 6). This observation suggests that lower ACC–DS connectivity is associated with improved behavioral performance and the ability of young rats to suppress a response bias and correctly perform the WM/BAT. In support of this notion, it has been reported that *Arc* expression in the DS of young mice decreases between early and late learning as mice adopt an optimal strategy (Pooters et al., 2017). Thus, the higher levels of *Arc* expression in the aged DS could be related to enhanced connectivity with the ACC and to the aged animals not switching from a response-based to an object-in-place strategy. Alternatively, it is also conceivable that the elevated expression of *Arc* in the DS of aged rats during behavior was related to altered reward experience. Young animals received more rewards than aged rats during the WM/BAT, but not during the control alternation task (Fig. 9*a*). Importantly, the young and aged rats had similar overlap in the active neural ensembles across tasks, suggesting that the difference in reward experiences across tasks and age groups is unlikely to fully account for elevated *Arc* expression in aged rats during WM/BAT performance.

The ACC directly projects to the DS (Gabbott et al., 2005; Fillinger et al., 2018), and both structures are also indirectly connected through the central medial nucleus of the thalamus (Vertes et al., 2012). The increased ACC–DS connectivity with training in aged rats may reflect impaired suppression of response-based strategies. This idea is consistent with multiple lines of evidence. First, it is widely reported that aged animals with hippocampal-dependent spatial memory impairments tend to overuse of response-based strategies (Barnes et al., 1980; Tomas Pereira et al., 2015) that are supported by the DS (Packard and McGaugh, 1992; Graybiel, 1998; Pych et al., 2005). Second, successful performance on the WM/BAT requires animals to flexibly update their behavior based on their position in the maze. Set shifting is compromised in aged rats (Barense et al., 2002; Beas et al., 2013, 2017), and this deficit has been linked to age-associated neurobiological alterations in the ACC and DS (Nicolle and Baxter, 2003; Nieves-Martinez et al., 2012). Finally, it is striking that the aged rats with less flexible and more response-driven behavior had high cellular *Arc* activity levels in the DS during performance of the WM/BAT, but not during the control spatial alternation task. It is known that neurons in DS that express *Arc* are GABAergic principal cells that are also positive for CaMKII (Vazdarjanova et al., 2006). A previous study showed that stochastic spatial exploration induces ∼5% of DS neurons to express *Arc* in young rats (Vazdarjanova et al., 2006). The current study adds to our understanding of *Arc* in DS by showing that when animals use response-driven behaviors, as the aged rats did during WM/BAT performance, there is an increased engagement of DS neurons. Together, these multiscale imaging data therefore suggest that the DS may be overactive during cognitive training in association with response-driven behaviors. Enhanced DS activity during behavior could be related to a reduced ability of the ACC to inhibit the DS, which then manifests as enhanced ACC–DS resting-state connectivity and perseverative suboptimal behavior.

An additional novel finding from the current data are the observation that rich-club organization changed over cognitive training in aged, but not young, rats. The baseline rich-club indices for nodes with high edge values (*k* > 27) were lower in aged compared with young rats (Fig. 7*a*). This observation is consistent with data from human study participants who have reported less functional rich-club participation in older adults compared with younger adults (Cao et al., 2014a). As in previous studies (Liang et al., 2018), the young rats did not show a change in rich-club participation across cognitive training (Fig. 7*b*). In contrast, the aged rats had a significant increase in rich-club participation between baseline and the second scan. This increase persisted in the third scan, although the aged rats displayed little to no improvements across the 25 d of cognitive testing (Fig. 1*b*). These data are consistent with reports of network connectivity measures in humans, as a previous longitudinal study reported that older adults had less network stability over time compared with young study participants (Iordan et al., 2017). Moreover, enhanced rich-club participation in aged rats could reflect a dedifferentiation of brain networks that has been observed in humans (Chan et al., 2014; Geerligs et al., 2015).

Rich-club organization and the strength and proportion of long-distance connections are hypothesized to play a central role in optimizing global brain communication efficiency for normal cognition (Bullmore and Sporns, 2009, 2012; van den Heuvel et al., 2012). Presumably, at the foundation of the functional rich club are hub neurons that have long-range projections. Interestingly, recent data have suggested that these neurons may be particularly vulnerable in advanced age, with subsets of them being over-recruited during behavior in aged rats (Hernandez et al., 2018b). An over-recruitment of vulnerable brain networks in aged animals during behavior could manifest as enhanced resting-state rich-club participation that ultimately reflects less adaptive networks and a reduced ability to recruit additional resources during behavior to improve performance. In line with this notion are recent data showing that higher levels of neural activity in prefrontal areas do not carry additional information related to cognitive performance (Morcom and Henson, 2018). In fact, elevated resting-state connectivity could be related to reduced neural efficiency (Park et al., 2004; Nyberg et al., 2012) or a dedifferentiation in older networks (Carp et al., 2011; Hoffman and Morcom, 2018).

While the increases in connectivity and rich-club participation observed in the current study could be consistent with age-related neural inefficiency, we cannot rule out the possibility that activity differences in the aged rats are compensatory. While all old rats performed poorly at a WM/BAT relative to the young rats, aged and young rats were able to alternate similarly showing comparable performance during the working memory component of the task. It is conceivable that this aspect of the behavior was facilitated by the functional connectome reorganization that occurred in the aged rats between the first and later scans. The aged rats, however, were less able to recruit additional resources as cognitive load increase. This interpretation of the data would be consistent with the Compensation-Related Utilization of Neural Circuits hypothesis (CRUNCH). CRUNCH postulates that more neural resources are recruited by older adults during tasks with minimal cognitive load. This increased activation, could serve to compensate for a network that is compromised. As tasks become more difficult, the limits of network capacity may be reached in older adults and these compensatory mechanisms are no longer effective, leading to equivalent or less activation in older adults relative to young adults (Reuter-Lorenz and Cappell, 2008; Grady, 2012). In the current experiments, the older rats showed a quick increase in rich-club participation even when they were unable to correctly multitask, suggesting that network limits in older rats are reached faster. In fact, these data, along with a recent cellular imaging study showing elevated *Arc* expression in the medial prefrontal cortices of aged rats at rest (Hernandez et al., 2018b), indicate that baseline brain connectivity of older animals may be at close to maximum capacity with no cognitive load. If the capacity of a network to respond to increasing cognitive load is constrained by increased rich-club participation, then aged rats may be less able to recruit additional resources when cognitively multitasking. Ultimately, this elevated rich-club participation in old animals could contribute to the reduced dynamic range of neural activity that has been reported for older adults (Kennedy et al., 2017).

An important impact of higher rich-club participation and resting-state connectivity in aged rats could be to tax brain energy reserves that may already be compromised in advanced age (Yoshizawa et al., 2014; Goyal et al., 2017; Hernandez et al., 2018a). Densely connected, long-distance projections are metabolically costly (Bullmore and Sporns, 2012). The higher costs associated with functional connections across multiple hubs makes them particularly vulnerable to metabolic deficiencies and cellular dysfunction contributing to instability in older animals. Thus, an enticing hypothesis is that improving the metabolic capacity of older animals could restore the dynamic range and functional rich-club architecture. In the future, large-scale assessment of network connectivity in conjunction with single-neuron activity dynamics and metabolic function could elucidate productive therapeutic avenues for treating cognitive aging.

## Synthesis

Reviewing Editor: Mark Baxter, Mount Sinai School of Medicine Decisions are customarily a result of the Reviewing Editor and the peer reviewers coming together and discussing their recommendations until a consensus is reached. When revisions are invited, a fact-based synthesis statement explaining their decision and outlining what is needed to prepare a revision will be listed below. The following reviewer(s) agreed to reveal their identity: NONE.

This manuscript was accepted after editorial review.
